# Bis[μ-4-hydr­oxy-*N*′-(4-meth­oxy-2-oxido­benzyl­idene)benzohydrazidato]bis­[pyridine­copper(II)]

**DOI:** 10.1107/S1600536810011323

**Published:** 2010-03-31

**Authors:** Nooraziah Mohd Lair, Hamid Khaledi, Hapipah Mohd Ali, Rustam Puteh

**Affiliations:** a Department of Chemistry, University of Malaya, 50603 Kuala Lumpur, Malaysia; b Department of Physics, University of Malaya, 50603 Kuala Lumpur, Malaysia

## Abstract

In the title compound, [Cu_2_(C_15_H_12_N_2_O_4_)_2_(C_6_H_5_N)_2_], each Cu^II^ atom is chelated by the tridentate doubly deprotonated Schiff base and a pyridine mol­ecule in a nearly planar environment (r.m.s. deviation for all non-H atoms = 0.107 Å). The metal ions are bridged by one O atom from the symmetry-related Schiff base ligands, forming a centrosymmetric dinuclear copper(II) complex. The dimeric complex is linked to another dimer *via* weaker Cu—O inter­actions and also O—H⋯N hydrogen bonds.

## Related literature

For the crystal structure of the monohydrated Schiff base ligand, see: Mohd Lair *et al.* (2009*a* ▶). For the structure of the pyridine adduct of the copper complex of the 4-nitro analog, see: Mohd Lair *et al.* (2009*b* ▶). For the crystal structure of a dinuclear copper(II) salphen complex with a similar coordin­ation, see: Yu *et al.* (2008 ▶).
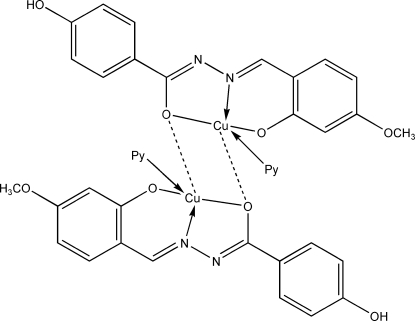


## Experimental

### 

#### Crystal data

[Cu_2_(C_15_H_12_N_2_O_4_)_2_(C_6_H_5_N)_2_]
*M* *_r_* = 853.83
Monoclinic, 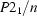

*a* = 13.3666 (3) Å
*b* = 7.9402 (2) Å
*c* = 16.7229 (3) Å
β = 94.775 (1)°
*V* = 1768.70 (7) Å^3^
*Z* = 2
Mo *K*α radiation
μ = 1.27 mm^−1^
*T* = 100 K
0.26 × 0.12 × 0.01 mm

#### Data collection

Bruker SMART APEXII diffractometer
Absorption correction: multi-scan (*SADABS*; Sheldrick, 1996 ▶) *T*_min_ = 0.734, *T*_max_ = 0.991
10121 measured reflections
4035 independent reflections
3473 reflections with *I* > 2σ(*I*)
*R*_int_ = 0.021

#### Refinement

*R*[*F* ^2^ > 2σ(*F* ^2^)] = 0.030
*wR*(*F* ^2^) = 0.075
*S* = 1.01
4035 reflections
257 parameters
1 restraint
H atoms treated by a mixture of independent and constrained refinement
Δρ_max_ = 0.41 e Å^−3^
Δρ_min_ = −0.26 e Å^−3^

### 

Data collection: *APEX2* (Bruker, 2007 ▶); cell refinement: *SAINT* (Bruker, 2007 ▶); data reduction: *SAINT*; program(s) used to solve structure: *SHELXS97* (Sheldrick, 2008 ▶); program(s) used to refine structure: *SHELXL97* (Sheldrick, 2008 ▶); molecular graphics: *X-SEED* (Barbour, 2001 ▶); software used to prepare material for publication: *publCIF* (Westrip, 2010 ▶).

## Supplementary Material

Crystal structure: contains datablocks I, global. DOI: 10.1107/S1600536810011323/om2326sup1.cif

Structure factors: contains datablocks I. DOI: 10.1107/S1600536810011323/om2326Isup2.hkl

Additional supplementary materials:  crystallographic information; 3D view; checkCIF report

## Figures and Tables

**Table 1:** Hydrogen-bond geometry (Å, °)

*D*—H⋯*A*	*D*—H	H⋯*A*	*D*⋯*A*	*D*—H⋯*A*
O1—H1⋯N1^i^	0.83 (1)	1.91 (1)	2.743 (2)	178 (3)

Symmetry code: (i) 
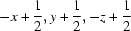
.

**Fig. 1.**
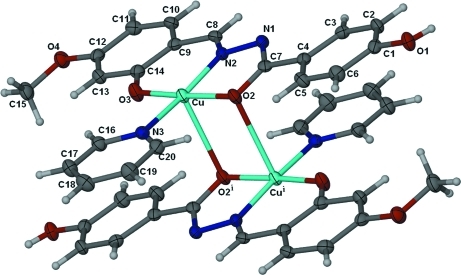
Thermal ellipsoid plot of the title compound at 60% probability level. Hydrogen atoms are drawn as spheres of arbitrary radius.

**Fig. 2.**
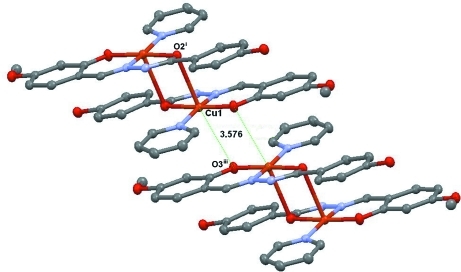
A view of the interaction between Cu1 and O3 from the symmetry related molecule at (-x+1, -y+1, -z). Hydrogen atoms have been omitted for clarity.

## supplementary crystallographic information

### Comment

The title compound is the pyridine adduct of the copper complex of 4-hydroxy-*N*'-(2-hydroxy-4-methoxybenzylidene)benzohydrazide. In the asymmetric unit, which contains one half of the formula unit, the copper ion is four coordinated in an approximately planar environment, the highest deviation from the best plane passing through all non-H atoms being 0.348 (2) Å for O2. From this point of view, it is similar to the structure of the 4-nitrated analogous compound (Mohd Lair *et al.* 2009*b*). However, replacement of the electron-withdrawing nitro group by a hydroxy group resulted in bridging the copper ions by O2 atoms from the symmetry related Schiff bases at (-x+1, -y+2, -z), forming a centrosymmetric dinuclear Cu^II^ complex. The distance of Cu1—O2^i ^is 2.778 (1) Å which is similar to the length of the Cu—O bridge (2.783 Å) in the dinuclear copper (II) salphen complex (Yu *et al.* 2008). Morever, there is a weak interaction between the copper ions and O3 atoms from the symmetry related molecules at (-x+1, -y+1, -z) with Cu1—O3^iii ^distance of 3.576 (2) Å, which binds the molecules in one-dimensional infinite chains. Intermolecular hydrogen bonds between the hydroxy groups and the imine N atoms of the neighboring molecules connect the complexes to each other.

### Experimental

The Schiff base ligand was prepared as reported previously (Mohd Lair*et al., *2009a). A mixture of the Schiff base (0.57 g, 2 mmol) and copper(II) acetate monohydrate (0.4 g, 2 mmol) in the presence of a few drops of triethylamine was refluxed in ethanol (100 ml) for 5 hours. The resulting green precipitate was then filtered, washed with ethanol and dried over silica gel. The green crystal of the title compound was obtained by slow evaporation of a pyridine solution of the compound.

### Refinement

A low angle reflection, (-1 0 1), probably affected by extinction, was omitted from the dataset. C-bound hydrogen atoms were placed at calculated positions (C–H 0.95–0.98 Å), and were treated as riding on their parent atoms, with U(H) set to 1.2–1.5 times Ueq(C). The hydroxy H-atom was located in a difference Fourier map, and was refined with distance restraints of O–H 0.84±0.01 Å.

### Crystal data

**Table:**

[Cu_2_(C_15_H_12_N_2_O_4_)_2_(C_6_H_5_N)_2_]	*F*(000) = 876
*M**_r_* = 853.83	*D*_x_ = 1.603 Mg m^−^^3^
Monoclinic, *P*2_1_/*n*	Mo *K*α radiation, λ = 0.71073 Å
Hall symbol: -P 2yn	Cell parameters from 4307 reflections
*a* = 13.3666 (3) Å	θ = 2.8–30.2°
*b* = 7.9402 (2) Å	µ = 1.27 mm^−^^1^
*c* = 16.7229 (3) Å	*T* = 100 K
β = 94.775 (1)°	Lath, pale green
*V* = 1768.70 (7) Å^3^	0.26 × 0.12 × 0.01 mm
*Z* = 2	

### Data collection

**Table:**

Bruker SMART APEXII diffractometer	4035 independent reflections
Radiation source: fine-focus sealed tube	3473 reflections with *I* > 2σ(*I*)
graphite	*R*_int_ = 0.021
φ and ω scans	θ_max_ = 27.5°, θ_min_ = 2.0°
Absorption correction: multi-scan (*SADABS*; Sheldrick, 1996)	*h* = −17→17
*T*_min_ = 0.734, *T*_max_ = 0.991	*k* = −7→10
10121 measured reflections	*l* = −21→21

### Refinement

**Table:**

Refinement on *F*^2^	Primary atom site location: structure-invariant direct methods
Least-squares matrix: full	Secondary atom site location: difference Fourier map
*R*[*F*^2^ > 2σ(*F*^2^)] = 0.030	Hydrogen site location: inferred from neighbouring sites
*wR*(*F*^2^) = 0.075	H atoms treated by a mixture of independent and constrained refinement
*S* = 1.01	*w* = 1/[σ^2^(*F*_o_^2^) + (0.0332*P*)^2^ + 1.6174*P*] where *P* = (*F*_o_^2^ + 2*F*_c_^2^)/3
4035 reflections	(Δ/σ)_max_ = 0.001
257 parameters	Δρ_max_ = 0.41 e Å^−^^3^
1 restraint	Δρ_min_ = −0.26 e Å^−^^3^

### Special details

**Table:**

Geometry. All esds (except the esd in the dihedral angle between two l.s. planes) are estimated using the full covariance matrix. The cell esds are taken into account individually in the estimation of esds in distances, angles and torsion angles; correlations between esds in cell parameters are only used when they are defined by crystal symmetry. An approximate (isotropic) treatment of cell esds is used for estimating esds involving l.s. planes.
Refinement. Refinement of *F*^2^ against ALL reflections. The weighted *R*-factor wR and goodness of fit *S* are based on *F*^2^, conventional *R*-factors *R* are based on *F*, with *F* set to zero for negative *F*^2^. The threshold expression of *F*^2^ > σ(*F*^2^) is used only for calculating *R*-factors(gt) etc. and is not relevant to the choice of reflections for refinement. *R*-factors based on *F*^2^ are statistically about twice as large as those based on *F*, and *R*- factors based on ALL data will be even larger.

### Fractional atomic coordinates and isotropic or equivalent isotropic displacement parameters (Å^2^)

**Table:**

	*x*	*y*	*z*	*U*_iso_*/*U*_eq_	
Cu1	0.511594 (16)	0.77247 (3)	−0.003528 (13)	0.01666 (8)	
O1	0.36136 (10)	1.26026 (19)	0.39906 (8)	0.0223 (3)	
H1	0.3017 (9)	1.277 (3)	0.4068 (15)	0.033*	
O2	0.49690 (10)	0.92300 (18)	0.08564 (8)	0.0189 (3)	
O3	0.51275 (10)	0.62681 (19)	−0.09181 (8)	0.0231 (3)	
O4	0.39119 (10)	0.28970 (18)	−0.31267 (8)	0.0226 (3)	
N1	0.33448 (12)	0.8237 (2)	0.07305 (9)	0.0165 (3)	
N2	0.37010 (11)	0.74438 (19)	0.00604 (9)	0.0159 (3)	
N3	0.66146 (12)	0.7893 (2)	0.00376 (9)	0.0169 (3)	
C1	0.36838 (14)	1.1762 (2)	0.32891 (11)	0.0178 (4)	
C2	0.28481 (14)	1.1087 (2)	0.28451 (11)	0.0191 (4)	
H2	0.2200	1.1206	0.3032	0.023*	
C3	0.29600 (14)	1.0246 (2)	0.21334 (11)	0.0178 (4)	
H3	0.2385	0.9800	0.1834	0.021*	
C4	0.39081 (14)	1.0044 (2)	0.18485 (11)	0.0166 (4)	
C5	0.47366 (14)	1.0747 (3)	0.22963 (11)	0.0207 (4)	
H5	0.5385	1.0642	0.2108	0.025*	
C6	0.46282 (14)	1.1589 (3)	0.30079 (11)	0.0213 (4)	
H6	0.5200	1.2051	0.3305	0.026*	
C7	0.40775 (14)	0.9119 (2)	0.11040 (10)	0.0165 (4)	
C8	0.30712 (14)	0.6628 (2)	−0.04221 (11)	0.0171 (4)	
H8	0.2384	0.6637	−0.0316	0.021*	
C9	0.33470 (14)	0.5707 (2)	−0.11095 (11)	0.0168 (4)	
C10	0.25867 (14)	0.4850 (2)	−0.15879 (11)	0.0192 (4)	
H10	0.1914	0.4927	−0.1448	0.023*	
C11	0.27833 (14)	0.3915 (3)	−0.22433 (11)	0.0206 (4)	
H11	0.2258	0.3346	−0.2552	0.025*	
C12	0.37823 (14)	0.3815 (2)	−0.24505 (11)	0.0188 (4)	
C13	0.45461 (14)	0.4615 (3)	−0.20009 (11)	0.0197 (4)	
H13	0.5214	0.4524	−0.2151	0.024*	
C14	0.43541 (14)	0.5572 (2)	−0.13177 (11)	0.0182 (4)	
C15	0.48858 (15)	0.2908 (3)	−0.34204 (12)	0.0251 (4)	
H15A	0.5092	0.4073	−0.3509	0.038*	
H15B	0.4865	0.2286	−0.3928	0.038*	
H15C	0.5368	0.2373	−0.3026	0.038*	
C16	0.71550 (15)	0.7054 (3)	−0.04781 (11)	0.0201 (4)	
H16	0.6810	0.6402	−0.0890	0.024*	
C17	0.81867 (15)	0.7103 (3)	−0.04325 (12)	0.0239 (4)	
H17	0.8543	0.6481	−0.0803	0.029*	
C18	0.87010 (15)	0.8063 (3)	0.01561 (12)	0.0249 (4)	
H18	0.9413	0.8124	0.0196	0.030*	
C19	0.81467 (15)	0.8934 (3)	0.06856 (12)	0.0249 (4)	
H19	0.8475	0.9612	0.1095	0.030*	
C20	0.71148 (15)	0.8808 (3)	0.06130 (11)	0.0207 (4)	
H20	0.6744	0.9394	0.0986	0.025*	

### Atomic displacement parameters (Å^2^)

**Table:**

	*U*^11^	*U*^22^	*U*^33^	*U*^12^	*U*^13^	*U*^23^
Cu1	0.01512 (12)	0.01935 (13)	0.01608 (12)	0.00010 (9)	0.00468 (8)	−0.00223 (9)
O1	0.0198 (7)	0.0291 (8)	0.0184 (7)	0.0027 (6)	0.0046 (5)	−0.0057 (6)
O2	0.0171 (6)	0.0236 (7)	0.0171 (6)	−0.0008 (6)	0.0066 (5)	−0.0034 (6)
O3	0.0162 (6)	0.0304 (8)	0.0231 (7)	−0.0017 (6)	0.0048 (5)	−0.0092 (6)
O4	0.0235 (7)	0.0255 (8)	0.0196 (7)	−0.0019 (6)	0.0061 (5)	−0.0054 (6)
N1	0.0192 (7)	0.0173 (8)	0.0139 (7)	0.0010 (6)	0.0069 (6)	0.0004 (6)
N2	0.0175 (7)	0.0155 (8)	0.0154 (7)	0.0010 (6)	0.0063 (6)	0.0004 (6)
N3	0.0175 (7)	0.0172 (8)	0.0162 (7)	0.0013 (6)	0.0031 (6)	0.0023 (6)
C1	0.0223 (9)	0.0170 (9)	0.0147 (8)	0.0032 (7)	0.0045 (7)	0.0009 (7)
C2	0.0159 (9)	0.0220 (10)	0.0204 (9)	0.0018 (7)	0.0068 (7)	−0.0001 (8)
C3	0.0176 (9)	0.0166 (10)	0.0195 (9)	−0.0011 (7)	0.0029 (7)	−0.0005 (7)
C4	0.0194 (9)	0.0156 (9)	0.0154 (8)	0.0015 (7)	0.0041 (7)	0.0024 (7)
C5	0.0169 (9)	0.0262 (11)	0.0196 (9)	0.0003 (8)	0.0051 (7)	0.0005 (8)
C6	0.0175 (9)	0.0262 (11)	0.0202 (9)	−0.0003 (8)	0.0015 (7)	−0.0016 (8)
C7	0.0196 (9)	0.0158 (10)	0.0147 (8)	0.0019 (7)	0.0055 (7)	0.0043 (7)
C8	0.0176 (9)	0.0153 (9)	0.0192 (9)	−0.0003 (7)	0.0064 (7)	0.0034 (7)
C9	0.0201 (9)	0.0141 (9)	0.0165 (8)	−0.0002 (7)	0.0038 (7)	0.0018 (7)
C10	0.0167 (9)	0.0196 (10)	0.0219 (9)	−0.0007 (7)	0.0059 (7)	0.0022 (8)
C11	0.0202 (9)	0.0212 (10)	0.0202 (9)	−0.0032 (8)	0.0015 (7)	−0.0002 (8)
C12	0.0248 (10)	0.0155 (9)	0.0166 (9)	0.0006 (7)	0.0054 (7)	0.0010 (7)
C13	0.0169 (9)	0.0217 (10)	0.0213 (9)	0.0006 (7)	0.0058 (7)	−0.0014 (8)
C14	0.0204 (9)	0.0164 (9)	0.0182 (9)	0.0001 (7)	0.0042 (7)	0.0013 (7)
C15	0.0250 (10)	0.0289 (11)	0.0224 (10)	0.0032 (9)	0.0084 (8)	−0.0036 (9)
C16	0.0221 (9)	0.0211 (10)	0.0176 (9)	−0.0010 (8)	0.0048 (7)	−0.0016 (8)
C17	0.0206 (9)	0.0296 (11)	0.0225 (9)	0.0012 (8)	0.0074 (8)	−0.0028 (8)
C18	0.0184 (9)	0.0343 (12)	0.0221 (9)	−0.0018 (9)	0.0027 (7)	0.0000 (9)
C19	0.0231 (10)	0.0305 (12)	0.0209 (9)	−0.0016 (9)	0.0006 (8)	−0.0048 (8)
C20	0.0222 (9)	0.0219 (10)	0.0184 (9)	0.0018 (8)	0.0043 (7)	−0.0005 (8)

### Geometric parameters (Å, °)

**Table:**

Cu1—O3	1.8765 (14)	C5—H5	0.9500
Cu1—N2	1.9242 (15)	C6—H6	0.9500
Cu1—O2	1.9338 (13)	C8—C9	1.436 (3)
Cu1—N3	2.0012 (16)	C8—H8	0.9500
Cu1—O2^i^	2.7784 (14)	C9—C10	1.414 (3)
O1—C1	1.360 (2)	C9—C14	1.422 (3)
O1—H1	0.830 (10)	C10—C11	1.367 (3)
O2—C7	1.297 (2)	C10—H10	0.9500
O3—C14	1.306 (2)	C11—C12	1.409 (3)
O4—C12	1.368 (2)	C11—H11	0.9500
O4—C15	1.429 (2)	C12—C13	1.372 (3)
N1—C7	1.318 (2)	C13—C14	1.413 (3)
N1—N2	1.403 (2)	C13—H13	0.9500
N2—C8	1.291 (2)	C15—H15A	0.9800
N3—C20	1.340 (2)	C15—H15B	0.9800
N3—C16	1.346 (2)	C15—H15C	0.9800
C1—C6	1.390 (3)	C16—C17	1.375 (3)
C1—C2	1.396 (3)	C16—H16	0.9500
C2—C3	1.384 (3)	C17—C18	1.382 (3)
C2—H2	0.9500	C17—H17	0.9500
C3—C4	1.400 (3)	C18—C19	1.386 (3)
C3—H3	0.9500	C18—H18	0.9500
C4—C5	1.400 (3)	C19—C20	1.378 (3)
C4—C7	1.479 (2)	C19—H19	0.9500
C5—C6	1.383 (3)	C20—H20	0.9500
			
O3—Cu1—N2	93.82 (6)	N2—C8—C9	123.94 (17)
O3—Cu1—O2	174.65 (6)	N2—C8—H8	118.0
N2—Cu1—O2	81.02 (6)	C9—C8—H8	118.0
O3—Cu1—N3	90.87 (6)	C10—C9—C14	118.39 (17)
N2—Cu1—N3	171.29 (6)	C10—C9—C8	118.51 (16)
O2—Cu1—N3	94.43 (6)	C14—C9—C8	123.06 (17)
O3—Cu1—O2^i^	98.64 (5)	C11—C10—C9	122.52 (17)
N2—Cu1—O2^i^	98.16 (5)	C11—C10—H10	118.7
O2—Cu1—O2^i^	80.83 (5)	C9—C10—H10	118.7
N3—Cu1—O2^i^	88.37 (5)	C10—C11—C12	118.48 (18)
C1—O1—H1	110.6 (18)	C10—C11—H11	120.8
C7—O2—Cu1	111.04 (12)	C12—C11—H11	120.8
C14—O3—Cu1	127.26 (12)	O4—C12—C13	124.03 (17)
C12—O4—C15	117.55 (15)	O4—C12—C11	114.89 (17)
C7—N1—N2	109.19 (14)	C13—C12—C11	121.07 (17)
C8—N2—N1	118.52 (15)	C12—C13—C14	120.99 (17)
C8—N2—Cu1	126.91 (13)	C12—C13—H13	119.5
N1—N2—Cu1	114.57 (11)	C14—C13—H13	119.5
C20—N3—C16	117.79 (17)	O3—C14—C13	116.87 (16)
C20—N3—Cu1	121.15 (13)	O3—C14—C9	124.60 (17)
C16—N3—Cu1	121.04 (13)	C13—C14—C9	118.53 (17)
O1—C1—C6	118.04 (17)	O4—C15—H15A	109.5
O1—C1—C2	122.52 (16)	O4—C15—H15B	109.5
C6—C1—C2	119.43 (17)	H15A—C15—H15B	109.5
C3—C2—C1	120.25 (17)	O4—C15—H15C	109.5
C3—C2—H2	119.9	H15A—C15—H15C	109.5
C1—C2—H2	119.9	H15B—C15—H15C	109.5
C2—C3—C4	120.92 (17)	N3—C16—C17	122.65 (18)
C2—C3—H3	119.5	N3—C16—H16	118.7
C4—C3—H3	119.5	C17—C16—H16	118.7
C3—C4—C5	118.07 (17)	C16—C17—C18	119.48 (18)
C3—C4—C7	123.35 (17)	C16—C17—H17	120.3
C5—C4—C7	118.58 (16)	C18—C17—H17	120.3
C6—C5—C4	121.20 (17)	C17—C18—C19	118.02 (19)
C6—C5—H5	119.4	C17—C18—H18	121.0
C4—C5—H5	119.4	C19—C18—H18	121.0
C5—C6—C1	120.12 (18)	C20—C19—C18	119.46 (19)
C5—C6—H6	119.9	C20—C19—H19	120.3
C1—C6—H6	119.9	C18—C19—H19	120.3
O2—C7—N1	123.45 (16)	N3—C20—C19	122.60 (18)
O2—C7—C4	116.35 (16)	N3—C20—H20	118.7
N1—C7—C4	120.20 (16)	C19—C20—H20	118.7

Symmetry codes: (i) −*x*+1, −*y*+2, −*z*.

### Hydrogen-bond geometry (Å, °)

**Table:**

*D*—H···*A*	*D*—H	H···*A*	*D*···*A*	*D*—H···*A*
O1—H1···N1^ii^	0.83 (1)	1.91 (1)	2.743 (2)	178 (3)

Symmetry codes: (ii) −*x*+1/2, *y*+1/2, −*z*+1/2.
